# Exploring student satisfaction and acceptance of e-learning technologies in Saudi higher education

**DOI:** 10.3389/fpsyg.2022.939336

**Published:** 2022-10-10

**Authors:** Mohammed Ayid Alqahtani, Mahdi Mohammed Alamri, Amer Mutrik Sayaf, Waleed Mugahed Al-Rahmi

**Affiliations:** ^1^Department of Educational Technology, College of Education, University of Bisha, Bisha, Saudi Arabia; ^2^Educational Technologies, King Faisal University, Al Hofuf, Saudi Arabia; ^3^Faculty of Social Sciences and Humanities, School of Education, Universiti Teknologi Malaysia, Skudai, Malaysia

**Keywords:** e-learning system, TAM model, structural equations modeling (SEM), higher education, research model development

## Abstract

The COVID-19 pandemic has rekindled interest in online learning as a desirable substitute. In Saudi Arabia’s educational system, technology and online learning are becoming more and more significant. In order to prepare students for the digital age and Saudi Vision 2030, there is an increasing desire for educational institutions to use e-learning. Students and faculty at Saudi institutions now have more opportunities to better grasp the globalized digital age thanks to the integration and acceptance of digital technology into learning and teaching. Therefore, this study aims to analyze and investigate the educational quality, social influence, and TAM Model factors that increase the students’ attitude toward using e-learning; thus, it affects students’ satisfaction and academic performance. The study was conducted at two universities in Saudi Arabia. Structural equation modeling (SEM) and route analysis were used to evaluate the research model and analyze data from e-learning users through a questionnaire. The findings revealed that perceived ease of use (PEU) and perceived usefulness (PU) mediate the effects of educational quality (EDQ), social influence (SOI), and perceived enjoyment (PE), which in turn affect students’ attitude toward use (ATU), and students’ satisfaction with using e-learning systems (SSE). Additionally, the results demonstrated that the mediator factors had favorable “R square (R^2^)” values for adopting e-learning systems in higher education, with PEU = 0.562, PU = 0.712, ATU = 0.608, and SSE = 0.636. The hypotheses’ findings led to the development of a validated instrument to measure students’ online learning in Saudi Arabia’s higher education.

## Introduction

E-learning is described as a “teaching and learning approach that completely or partially embodies the educational paradigm employed, and that aids in accepting novel ways of understanding and establishing learning based on the use of electronic media and devices as tools for enhancing the availability of training, communication, and interaction, and that aids in accepting novel ways of understanding and establishing learning”. E-learning is described as learning that takes place on a variety of electronic devices, and it is characterized as learning that takes place on a variety of electronic devices in today’s world. Computers, mobile phones, laptops, and virtual worlds are examples of computational devices (Lee et al., 2009). E-learning is increasingly becoming a vital tool that educational institutions and universities throughout the world are adopting (Kumar and Owston, 2016; Yeh and Chu, 2018). According to Al-Rahmi et al. (2021b), e-learning establishes a virtual environment where students may participate in a variety of activities. Using an e-learning system has several advantages. Only a few of the advantages (Bonk et al., 2004; Concannon et al., 2005) include quick access to material information, simple team interaction, and timely shared discussions. Thanks to the prevalence of physical infrastructure in developing countries, these advantages could be expanded much further. The regional divide may also be bridged. The e-learning system, on the other hand, has only been partially or completely accepted in developed countries; its implementation is incomplete and deemed unsatisfactory (Tarhini et al., 2017). This refers to a scarcity of data on the factors that influence its adoption (Al-rahmi et al., 2015b; Salloum et al., 2019). Furthermore, the majority of previous research has concentrated on establishing the influence of certain traits on e-learning utilization. These factors vary from research to research, depending on the subjects and the situation. The bulk of this research has concentrated on individual components of the primary drivers of e-learning system efficiency, neglecting the synergistic impact of success variables interacting with one another (Eom and Ashill, 2018). Other research, Selim (2003) and Ozkan and Koseler (2009) investigated the direct links between e-learning quality parameters and usage or satisfaction. A robust performance model for a wide range of achievement levels is necessary (Eom and Ashill, 2018). It’s critical to assess both human (learners and instructors) and non-human (e.g., learning management systems) participants in an e-learning system for success. Prior studies have tended to concentrate on the technology itself. Recent studies have highlighted the role of students’ and instructors’ attitudes and interactions in e-learning performance (Cheng, 2011; Liaw and Huang, 2013). As technology becomes more effective and available, recent research has emphasized the importance of students’ and instructors’ attitudes and interactions in e-learning success. So, more research is required to analyze these programs in order to develop them and address the needs of students. As a result, it is thought that a complete theoretical model is needed to fully comprehend the factors that influence e-learning adoption in any setting, regardless of background or individual. Thus, this manuscript’s major purpose is to fix the foregoing flaws in two ways. Firstly, we review the Technology Acceptance Model (TAM)-based e-learning research articles and identify the most common external factors. Second, by expanding TAM to include these variables, we can empirically analyze the influence of external factors that have provided major findings in the literature on students’ e-learning use. Identifying these factors may also help decision-makers recognize the strengths and weaknesses of e-learning infrastructure and increase technological acceptability.

### Problem background

Education is given top priority in Saudi Arabia. For more than 25 years (Alturki, 2014), Saudi Arabia has attempted to enhance the educational process by incorporating computers into the curriculum. The first university in Saudi Arabia to link to the internet was King Fahad University of Petroleum and Minerals in 1993. Saudi Arabia has developed a national plan to implement information technology in higher education by 2008 (Chanchary and Islam, 2011). As a result, implementing e-learning during the COVID-19 pandemic has not presented challenges for higher education. Since the COVID-19 epidemic, distance learning has gained strategic importance for educational systems around the globe, including those in the Kingdom of Saudi Arabia (Hebebci et al., 2020; Kim, 2020; Sun et al., 2020). This does not imply that Saudi Arabian educational institutions have not previously given distant learning programs any thought, as there have been several initiatives in this area (Alahmari, 2017). The COVID-19 pandemic has affected Saudi Arabia’s educational system in the same way that it has in practically every other nation on earth. It compelled practically all schools to switch to online learning methods in place of face-to-face meetings, rather than only some schools (Alammary et al., 2021). According to Alkhalaf et al. (2012), professors at King Abdulaziz University and Qassim University both showed favorable opinions regarding e-learning. Hoq (2020) looked at instructors’ preferences for e-learning in the Jubail Industrial College’s Management and Information Technology Department during the COVID-19 epidemic and discovered that they had positive sentiments toward it. According to Almaghaslah and Alsayari (2020), more than half of the academic staff of King Khalid University’s College of Pharmacy had favorable attitudes toward e-learning. Additionally, despite the fact that online education tends to encourage student autonomy, Saudi students’ engagement in online classes is frequently influenced by prior exposure to the country’s traditional method of teaching and training, in which teachers assume a central role and have complete control over students (Hamdan, 2014), which effectively limits opportunities for active online student participation (Lobo et al., 2020). Similar findings were made by Alhabeeb and Rowley (2017), who discovered that academic staff members play a crucial role in encouraging efficient e-learning in Saudi Arabian universities through their familiarity with educational technology, computer system users, and technological infrastructure.

## Research model and hypotheses development

**FIGURE 1:**
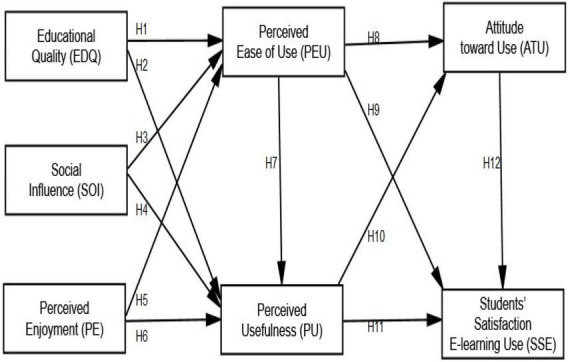
Research model.

The Technology Adoption Model was developed by Davis (1989), which has been employed in a number of research projects and has gotten a lot of attention in the literature on technology adoption (Chang et al., 2017). In a recent systematic study (Al-Qaysi et al., 2020; Ullah et al., 2021), TAM has also been shown to be effective in educational technology adoption when compared to other theoretical models (Hassanzadeh et al., 2012; Ullah et al., 2021). In higher education, the e-learning system has revolutionized teaching and learning. The adoption of studies has resulted in a plethora of complimentary and opposing models, the bulk of which are linked to the use of information systems (IS), such as e-learning. TAM is likely the most important theoretical contribution to adoption research, and academics use it to evaluate e-learning systems all the time. The current study looked at the following seven factors that influence e-learning uptake in higher education: The abbreviations EDQ, SOI, PE, PEU, PU, ATU, and SSE are shown in Figure 1.

### Educational quality

As a new dimension to the IS performance model proposed by Hassanzadeh et al. (2012), EDQ is regarded as device quality in terms of features and functionality it may provide to enhance users’ learning and training. In terms of collaborative learning, the extent to which an IS system is able to offer an appropriate learning environment for learners (Hassanzadeh et al., 2012; Kim et al., 2012) can be described as the EDQ. Hassanzadeh et al. (2012) found in their research that the EDQ has a favorable influence on individual satisfaction, which is also corroborated by Kim et al. (2012), who observed that the standard of training has a substantial beneficial impact on user satisfaction. As a result, it’s assumed that the EDQ has a favorable impact on individual satisfaction as well as intention to use. In a report on digital learning systems, the association between EDQ and PU was determined to be relevant for e-learning systems that are available over the internet in a report on digital learning systems by Liu et al. (2005) and Almaiah et al. (2016). EDQ and SSE discovered a relationship according to Kim et al. (2012) and Mohammadi (2015). Furthermore, Almaghaslah and Alsayari (2020) and Cidral et al. (2018) discovered substantial correlations between the diversity of evaluation resources, learner engagement, and e-learning system satisfaction. The following hypotheses were suggested based on the discussion above:

EDQ is positively with PEU.

EDQ is positively with PU.

### Social influence

The subjective standard’s pressure is represented by SOI, which is described as “the feeling of collective effect on an individual’s decision.” If key people in the company, such as managers, advocate the use of technology, users will think it is more successful in achieving job-related goals. In previous studies (Venkatesh et al., 2003; Abdullah and Ward, 2016; Dwivedi et al., 2017), social variables have been demonstrated to have a predictive influence on success anticipation (Venkatesh et al., 2003; Abdullah and Ward, 2016; Dwivedi et al., 2017). In Africa, research has revealed a variety of social influence effects on PEU and PU, which are similar to success expectations (Mtebe and Raisamo, 2014; Al-Rahmi et al., 2015c). Learners who see e-learning as being supported by others in their social environment are more likely to continue using it in the study’s settings. As a result, the VETA model, like the UTAUT and UTAUT2 models, includes SOI as a predictor of PEU and PU. The following hypotheses were suggested based on the discussion above:

SOI is positively with PEU.

SOI is positively with PU.

### Perceived enjoyment

According to Punnoose (2012), perceived enjoyment (PE) may be used to characterize the desire to acquire and use information systems. User acceptability and e-learning usage behavior are also influenced by a student’s subjective feelings of delight, relaxation, and enjoyment when studying, as well as a positive overall experience (Saadé et al., 2008). According to Venkatesh et al. (2003), people who find the technology they use intriguing learn to enjoy the task they are performing, comprehend its PU, and find it simpler to use. Because more technology is integrated into e-learning, it can be more enjoyable than traditional classroom learning. This was supported by a study conducted by van der Heijden (2004), which discovered that intrinsic motivators such as PE might affect a user’s inclination to use information systems such as e-learning; the findings demonstrated that PE had a substantial impact on student PU and PEU for e-learning. The following hypotheses were suggested based on the discussion above:

PE is positively with PEU.

PE is positively with PU.

### Perceived ease of use

The perceived ease of use (PEU) of a system is the degree to which someone believes that utilizing a specific technology is straightforward (Davis, 1989). In the past, there has been evidence of a positive relationship between PEU and ATU, both directly and indirectly (Alharbi and Drew, 2014; Tarhini et al., 2017). PEU refers to a student’s perception that using an e-learning system would take minimal time and be simple to use in the context of e-learning. In addition, earlier studies have discovered a substantial relationship between PEU and PU (Al-Gahtani, 2016; Abbas, 2017). In addition, past studies have discovered a link between PEU and attitudes about utilizing an e-learning system (Jung et al., 2008; Alharbi and Drew, 2014; Fathema et al., 2015). The following hypotheses were suggested based on the discussion above:

PEU is positively with PU.

PEU is positively with ATU.

PEU is positively with SSE.

### Perceived usefulness

According to Davis (1989), perceived usefulness (PU) is the degree to which individuals believe that employing current technology will improve their jobs’ efficiency. In several empirical investigations (Al-Busaidi, 2013; Tarhini et al., 2017), PU has been proven to be the most crucial element in determining whether or not to utilize a specific technology (Ozkan and Koseler, 2009). Students may only use the e-learning system if they feel it would help them improve their grades. According to previous e-learning studies (Al-Rahmi et al., 2015a; Mahmodi, 2017), PU and the ATU e-learning system have a significant and favorable association. According to the literature, there is considerable empirical support for a connection between PU and attitudes toward usage (Cheng, 2011; Al-Adwan et al., 2013; Alamri et al., 2020b). The following hypotheses were suggested based on the discussion above:

PU is positively with ATU.

PU is positively with SSE.

### Students’ attitude toward use

The way a person feels about e-learning systems and how good or bad that emotion is, is referred to as their attitude (Hussein, 2017). Several studies (Alharbi and Drew, 2014; Fathema et al., 2015) have discovered that attitude has a significant influence on behavioral purpose and pleasure. Students’ attitudes about e-learning are frequently impacted by system features. Pituch and Lee (2006) claim that if a method is user-friendly, pupils are more likely to adopt it. Their studies also demonstrate that systems that allow students to interact effectively while simultaneously providing access to course materials have an impact on students’ desire to utilize the system for learning and satisfaction. Students’ capacity to participate in online learning is also influenced by their prior computer skills, according to Selim (2007). Learner attitudes regarding the internet have a significant impact on student happiness, interest, and quality in an online learning environment (Yang and Lin, 2010). The following hypothesis was suggested based on the discussion above:

ATU is positively with SSE.

### Students’ satisfaction

Every business’s principal purpose, rather than selling, supplying, or servicing, is to meet the demands and satisfy the satisfaction of its consumers (Sanchez-Franco, 2009; Docimini and Palumbo, 2013). User satisfaction is more precisely characterized as how satisfied users are with information and support services (Petter et al., 2008), since satisfaction is defined as an individual’s judgment of how well their criteria, goals, and wishes have all been accomplished (Sanchez-Franco, 2009). According to the new IS performance model, device usage comes before customer satisfaction, which leads to more contentment, which leads to a greater desire to use (Petter et al., 2008). In various research (Petter et al., 2008; Hassanzadeh et al., 2012), satisfaction was found to have a considerable positive influence on the intention to utilize e-learning services (Petter et al., 2008; Hassanzadeh et al., 2012). Actual consumption has also been demonstrated to have a major impact on satisfaction. According to Hassanzadeh et al. (2012), satisfaction had a favorable influence on actual usage of an e-learning system in their study. As a result, contentment is projected to have a beneficial impact on both intentions and actual usage in this study.

## Research methodology

To assess theoretical models and hypotheses, quantitative approaches are applied, and this study used a quantitative analytical survey. The measurement items were created from a literature review and were designed to cover every aspect of the construction process. Expert comments on the items chosen to signify each construction were sought as a follow-up phase (Hair et al., 2017). E-learning systems have been promoted by several institutions, including those in Saudi Arabia. As a result, the purpose of this study is to use empirical research to construct a model for measuring students’ attitudes toward and satisfaction with using an e-learning system. The study’s sample comprised students with both undergraduate and graduate degrees who utilized e-learning. A five-point Likert scale was utilized for items including TAM components, model constructs, and demographic data, with one indicating strong disagreement and five suggesting strong agreement. The measurement model’s validity and reliability were evaluated using the Statistical Package for the Social Sciences (SPSS) and Structural Equation Modeling (AMOS-SEM). For the model’s goodness of fit, factor loadings were utilized to establish build validity, composite reliability, Cronbach’s α, and convergence validity, as stated by Jung et al. (2008). Cronbach’s α was found to be 0.931 based on standardized items. Table 1 shows the reliability coefficient (Cronbach’s α) for both the pilot and final test designs; all variables were found appropriate. For more details, see Table 1.

**TABLE 1:** Reliability test (pilot and final).

No	Factors	Code	Pilot test	Final test
1	Educational quality	EDQ	0.778	0.911
2	Social influence	SOI	0.742	0.932
3	Perceived enjoyment	PE	0.830	0.943
4	Perceived ease of use	PEU	0.779	0.899
5	Perceived usefulness	PU	0.792	0.907
6	Attitude toward use	ATU	0.802	0.919
7	Students’ satisfaction	SSE	0.812	0.911

### Data collection and sample characteristics

This study was conducted online from February to April 2021, while institutions were closed due to the COVID-19 epidemic. A survey instrument was designed and verified before the major data collection to examine parameters predicting student usage of an e-learning system. We randomly selected 446 individuals for the study, who were then entered into the SPSS package program. Postgraduate and undergraduate students at Saudi Arabia University were effective users of the e-learning system during the COVID-19 outbreak.

### Instruments of measurement

The measurement scales’ material validity was proven by the construction components employed in previous studies. There were two sections to the research questionnaire: EDQ questionnaire items were adapted from Hassanzadeh et al. (2012), and questionnaire components were used to obtain basic demographic data (gender, age, educational level, and specialization). SSE was derived from DeLone and McLean (2003), and PE, PEU, PU, and ATU were taken from Davis (1989). SSE was derived from DeLone and McLean (2003), while social influence was taken from Venkatesh et al. (2003). All of the instruments were purchased from a reputable supplier. As a consequence, the factors were assessed using multi-item measures based on prior research and self-report. On a five-point Likert scale, 1 signified “strongly disagree” and 5 meant “strongly agree.” Confirmatory factor analysis is performed to evaluate the model’s validity, and all of the items are included in Table 2.

**TABLE 2:** Measurement model, item loadings, construct reliability, and convergent validity.

Variables	Code	Loading	AVE	CR	CA	R^2^
Educational quality	EDQ1	0.863	0.558	0.883	0.836	0.000
	EDQ2	0.753				
	EDQ3	0.802				
	EDQ4	0.801				
Social influence	SOI1	0.733	0.632	0.873	0.790	0.000
	SOI2	0.742				
	SOI3	0.793				
	SOI4	0.824				
Perceived enjoyment	PE1	0.882	0.739	0.887	0.894	0.000
	PE2	0.903				
	PE3	0.884				
Perceived ease of use	PEU1	0.700	0.662	0.907	0.900	0.562
	PEU2	0.713				
	PEU3	0.744				
	PEU4	0.772				
	PEU5	0.804				
Perceived usefulness	PU1	0.772	0.762	0.877	0.885	0.712
	PU2	0.823				
	PU3	0.814				
	PU4	0.803				
	PU5	0.783				
Attitude toward use	ATU1	0.842	0.692	0.890	0.897	0.608
	ATU2	0.823				
	ATU3	0.823				
	ATU4	0.704				
Students’ satisfaction	SSE1	0.833	0.721	0.848	0.898	0.636
	SSE2	0.892				
	SSE3	0.891				
	SSE4	0.844				
	SSE5	0.764				

## Analysis and findings

Covariance-based structural equation modeling was used to test the conceptual model in the thesis (CB-SEM). There are several benefits of using CB-SEM (Wu and Wang, 2005). The parameters were estimated using the greatest likelihood (ML) method. The AMOS software and the CB-SEM technique were used to analyze the data (v.24). As methodological metrics, both the mathematical and structural models were assessed. The structural model considers how ICT might be used to evaluate digital learning hypotheses, whereas the measurement model considers construct reliability, validity, and overall model fitness.

### Information about the population

Table 3 shows the demographic information. 168 (37.7%) of the 446 usable questionnaires surveyed were from male respondents, while 278 (62.3%) were from female respondents. Moreover, 115 (25.8%) were 18–21 years old, 123 (27.6%) were 22–25 years old, 42 (9.4%) were 26–29 years old, 55 (12.3%) were 30–33 years old, and 111 (24.9%) were more than 34 years old. 239 (53.6%) respondents from Bisha University and 207 respondents (46.4%) from King Faisal University. At the next level of education, 258 (57.8%) were undergraduate students, and 188 (42.2%) were postgraduate students. Also, by type of study, 248 (55.6%) study full time, and 198 (44.4%) study part time. And the faculties of study: 170 (38.1%) from the faculty of education; 35 (7.8%) from the faculty of science; 88 (19.7%) from the faculty of art and humanities; 28 (6.3%) from the faculty of medical science; and 125 (28.0%) from the faculty of computer science. Time of actual use of e-learning 305 (68.4%) used e-learning for less than 5 years, 80 (17.9%) used e-learning between 5 and 10 years, and 61 (13.7%) used e-learning for more than 10 years. Finally, actual use of e-learning showed that 307 (68.8%) were used always, 131 (29.4%) were used some of the time, and 8 (1.8%) were not used.

**TABLE 3:** Demographic information.

Characteristics		*N*	%	Characteristics		*N*	%
Gender	Male	168	37.7	University	Bisha University	239	53.6
	Female	278	62.3		King Faisal University	207	46.4
Faculties	Education	170	38.1	Age	More 34 years	111	24.3
	Science	35	7.8		30–33 years	55	12.3
	Art and humanities	88	19.7		26–29 years	42	9.4
	Medical science	28	6.3		22–25 years	123	27.6
	Computer science	125	28.0		18–21 years	115	25.8
Level of education	Undergraduate	258	57.8	Type of study	Full time	248	55.6
	Postgraduate	188	42.2		Part time	198	44.4
Time of use ICT	Less 5 years	305	68.4	Use ICT	Always	307	68.8
	5–10 years	80	17.9		Some time	131	29.4
	More 10 years	61	13.7		Not’ use	8	1.8

### Reliability, validity, and measurement model measures

The SEM-AMOS measurement model for each notion has its own set of characteristics, such as reliability and validity. Using human CFA and model fitness indicators from the measuring model, the structural model was used to assess the strength of the connecting route. Table 2 lists the components of the measurement. Because the majority of the commodities exceed the 0.707 criteria, the data demonstrates that item dependability is not a concern (Hair et al., 2017). The constructs’ internal consistency was measured using composite reliability, which varied from 0.942 to 0.889 and was greater than the cut-off value of 0.70 (Hair et al., 2017). The average variance extracted (AVE) for the constructs varied from 0.709 to 0.592, which was higher than the convergent validity threshold of 0.50 (Hair et al., 2017). Cross-loading, the square-root of AVE (Hair et al., 2017), ASV, and MSV measurements were employed to establish discriminant validity. The diagonal value is greater than the values of the adjacent row and column numbers. In Table 4, the values that are bolded indicate a stronger connection between the structure and other structures. Similarly, the maximum shared variance (MSV) is less than the average shared variance (ASV), but more than the average absolute variance (AVE) (Table 4). As a result, the measurement variables are independent of each other.

**TABLE 4:** Discriminant validity.

Factors and items		AVE	MSV	ASV	PE	SOI	EDQ	PEU	PU	ATU	SSE
Perceived enjoyment	PE	0.943	0.011	0.240	**0.894**						
Social influence	SOI	0.608	0.117	0.032	0.324	**0.840**					
Educational quality	EDQ	0.682	0.200	0.231	0.487	0.274	**0.807**				
Perceived ease of use	PEU	0.899	0.092	0.059	0.327	0.283	0.284	**0.856**			
Perceived usefulness	PU	0.907	0.211	0.102	0.383	0.276	0.316	0.276	**0.836**		
Attitude toward use	ATU	0.919	0.210	0.033	0.517	0.290	0.461	0.280	0.318	**0.839**	
Students’ satisfaction e-learning use	SSE	0.911	0.177	0.105	0.458	0.273	0.504	0.263	0.305	0.446	**0.864**

### Evaluation of the model’s fit

**FIGURE 2:**
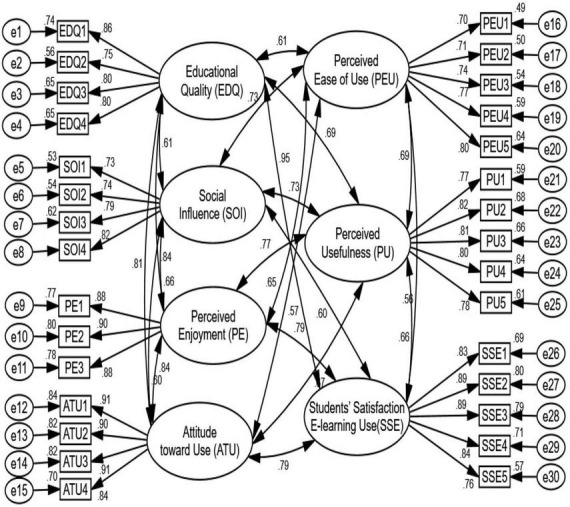
Model of measurement.

The CMN/DF value was 3.207, which was less than the cut-off threshold (5.00). IFI (0.942) is very good, GFI (0.955) is decent, CFI (0.937) is very good, and TLI (0.933) is very good. The RMR and RMSEA were both less than the thresholds of 0.31 (0.05) and 0.047 (0.08), respectively, indicating that the model’s badness measures were good (Hair et al., 2017). Figure 2 illustrates the whole set of data, proving that the measurement model was appropriate and well-suited to the structural model.

### Path coefficient and structural model

**FIGURE 3:**
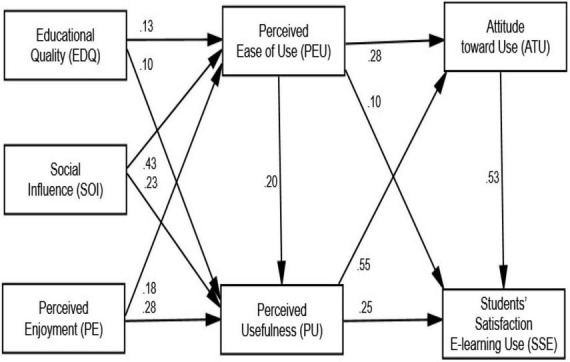
Structural model (*P* value).

The link between the independent and dependent variables is described by the route coefficient (path coefficients). The maximum likelihood approach may be used to evaluate intricate models and identify multiple links between multi-item variables as well as moderating and mediating effects (Berraies et al., 2017). The route coefficient is used in Figure 3 to show the direct effect of the latent predictor variable on predicted variables, see Table 5.

**TABLE 5:** Results of hypotheses testing.

Hypotheses and path				Beta (β)	Standard error	Critical ratio	*P* value	Results
Hypothesis 1	PEU	<—	EDQ	0.132	0.046	2.784	0.005	Supported
Hypothesis 2	PU	<—	EDQ	0.096	0.040	2.436	0.015	Supported
Hypothesis 3	PEU	<—	SOI	0.429	0.044	9.809	0.000	Supported
Hypothesis 4	PU	<—	SOI	0.233	0.042	5.599	0.000	Supported
Hypothesis 5	PEU	<—	PE	0.182	0.045	4.078	0.000	Supported
Hypothesis 6	PU	<—	PE	0.282	0.039	7.177	0.000	Supported
Hypothesis 7	PU	<—	PEU	0.198	0.041	4.835	0.000	Supported
Hypothesis 8	ATU	<—	PEU	0.280	0.055	5.042	0.000	Supported
Hypothesis 9	SSE	<—	PEU	0.100	0.044	2.295	0.022	Supported
Hypothesis 10	ATU	<—	PU	0.552	0.057	9.734	0.000	Supported
Hypothesis 11	SSE	<—	PU	0.250	0.048	5.234	0.000	Supported
Hypothesis 12	SSE	<—	ATU	0.530	0.036	14.613	0.000	Supported

## Factor description

The standard deviation (SD) and mean (mean) are two statistics that show how measurements deviate from the average (mean) or anticipated value in a population. The bulk of the data points are similar to the mean when the standard deviation is low. If the standard deviation is large, the data is more evenly dispersed. As a result, as shown in Tables 6–12, all ideals were approved, implying that university students who used e-learning improved their academic performance. Number (1) means “strongly disagree” number (2) means “disagree,” number (3) means “neutral,” number (4) means “agree,” and number (5) means “strongly agree.” According to the results in Table 6, the majority of students agree or strongly agree that educational quality has a positive influence on students’ using an e-learning system would help them learn more effectively.

**TABLE 6:** Measuring educational quality.

Factor and items		Numbers and percentages of respondents					Mean	S.D
		
		1	2	3	4	5		
Educational quality	EDQ1	6 (1.3%)	19 (4.3%)	58 (13.0%)	185 (41.5%)	178 (39.9%)	4.14	0.895
	EDQ2	6 (1.3%)	26 (5.8%)	99 (22.2%)	177 (39.7%)	138 (30.9%)	3.93	0.940
	EDQ3	4 (0.9%)	25 (5.6%)	54 (12.1%)	203 (45.5%)	160 (35.9%)	4.1	0.881
	EDQ4	8 (1.8%)	21 (4.7%)	68 (15.2%)	192 (43.0%)	157 (35.2%)	4.05	0.924

**TABLE 7:** Measuring social influence (SOI).

Factor and items		Numbers and percentages of respondents					Mean	S.D
		
		1	2	3	4	5		
Social influence	SOI1	3 (0.7%)	20 (4.5%)	55 (12.3%)	214 (48.0%)	145 (344.5%)	4.11	0.835
	SOI2	6 (1.3%)	22 (4.9%)	107 (24.0%)	187 (41.9%)	124 (27.8%)	3.9	0.91
	SOI3	3 (0.7%)	17 (3.8%)	78 (17.5%)	197 (44.2%)	151 (33.9%)	4.07	0.851
	SOI4	4 (0.9%)	2 (0.4%)	43 (9.6%)	192 (43.0%)	205 (46.0%)	4.33	0.741

**TABLE 8:** Measuring perceived enjoyment (PE).

Factor and items		Numbers and percentages of respondents					Mean	S.D
		
		1	2	3	4	5		
Perceived enjoyment	PE1	5 (1.1%)	16 (3.6%)	54 (12.1%)	184 (41.3%)	187 (41.9%)	4.19	0.866
	PE2	5 (1.1%)	18 (4.0%)	65 (14.6%)	188 (42.2%)	170 (38.1%)	4.12	0.881
	PE3	8 (1.8%)	25 (5.6%)	66 (14.8%)	173 (38.8%)	174 (39.0%)	4.08	0.959

**TABLE 9:** Measuring perceived ease of use (PEU).

Factor and items		Numbers and percentages of respondents					Mean	S.D
		
		1	2	3	4	5		
Perceived Ease of Use	PEU1	5 (1.1%)	7 (1.6%)	35 (7.8%)	197 (44.2%)	202 (45.3%)	4.31	0.775
	PEU2	6 (1.3%)	18 (4.0%)	68 (15.2%)	195 (43.7%)	159 (35.7%)	4.08	0.887
	PEU3	4 (0.9%)	14 (3.1%)	66 (14.8%)	194 (43.5%)	168 (37.7%)	4.14	0.845
	PEU4	3 (0.7%)	26 (5.8%)	46 (10.3%)	196 (43.9%)	175 (39.2%)	4.15	0.876
	PEU5	1 (0.2%)	22 (4.9%)	55 (12.3%)	185 (41.5%)	183 (41.0%)	4.18	0.848

**TABLE 10:** Measuring perceived usefulness (PU).

Factor and items		Numbers and percentages of respondents					Mean	S.D
		
		1	2	3	4	5		
Perceived Usefulness	PU1	1 (0.2%)	12 (2.7%)	40 (9.0%)	181 (40.6%)	212 (47.5%)	4.33	0.767
	PU2		8 (1.8%)	34 (7.6%)	186 (41.7%)	218 (48.9%)	4.38	0.704
	PU3	2 (0.4%)	13 (2.9%)	48 (10.8%)	182 (40.8%)	201 (45.1%)	4.27	0.802
	PU4	3 (0.7%)	19 (4.3%)	53 (11.9%)	183 (41.0%)	188 (42.2%)	4.20	0.857
	PU5	1 (0.2%)	16 (3.6%)	43 (9.6%)	182 (40.8%)	204 (%)45.7	4.28	0.799

**TABLE 11:** Measuring attitude toward use (ATU).

Factor and items		Numbers and percentages of respondents					Mean	S.D
		
		1	2	3	4	5		
Attitude toward use	ATU1	6 (1.3%)	14 (3.1%)	44 (9.9%)	178 (39.9%)	204 (45.7%)	4.26	0.860
	ATU2	5 (1.1%)	17 (3.8%)	43 (9.6%)	184 (41.3%)	197 (44.2%)	4.24	0.859
	ATU3	6 (1.3%)	15 (3.4%)	42 (9.4%)	184 (41.3%)	199 (44.6%)	4.24	0.859
	ATU4	10 (2.2%)	12 (2.7%)	49 (11.0%)	177 (39.7%)	198 (44.4%)	4.21	0.905

**TABLE 12:** Measuring students’ satisfaction with using e-learning systems (SSE).

Factor and items		Numbers and percentages of respondents					Mean	S.D
		
		1	2	3	4	5		
Students’ Satisfaction	SSE1	7 (1.6%)	14 (3.1%)	39 (8.7%)	181 (40.6%)	205 (46.0%)	4.26	0.864
	SSE2	9 (2.0%)	9 (2.0%)	52 (11.7%)	197 (44.2%)	179 (40.1%)	4.18	0.865
	SSE3	5 (1.1%)	20 (4.5%)	60 (13.5%)	196 (43.9%)	165 (37.0%)	4.11	0.880
	SSE4	8 (1.8%)	20 (4.5%)	48 (10.8%)	201 (45.1%)	169 (37.9%)	4.13	0.902
	SSE5	4 (0.9%)	11 (2.5%)	38 (8.5%)	207 (46.4%)	186 (41.7%)	4.26	0.786

According to the results in Table 7, the majority of students agree or strongly agree that social influence has a positive influence on students’ using an e-learning system would help them learn more effectively.

According to the results in Table 8, the majority of students agree or strongly agree that perceived enjoyment has a positive influence on students’ using an e-learning system would help them learn more effectively.

According to the results in Table 9, the majority of students agree or strongly agree that perceived ease of use has a positive influence on students’ using an e-learning system would help them learn more effectively.

According to the results in Table 10, the majority of students agree or strongly agree that perceived usefulness has a positive influence on students’ using an e-learning system would help them learn more effectively.

According to the results in Table 11, the majority of students agree or strongly agree that attitude toward use has a positive influence on students’ using an e-learning system would help them learn more effectively.

According to the results in Table 11, the majority of students agree or strongly agree that students’ satisfaction has a positive influence on their use of an e-learning system, which would help them learn more effectively.

## Discussion and implications

In this study, we predicted a link between the TAM Model, the EDQ, and the SOI in the context of e-learning use in higher education. The outcomes of the study add to the body of knowledge by demonstrating that students may enhance their e-learning by using the EDQ, SOI, and PE. The study also adds to the literature by establishing relationships between student SSE values and the EDQ, SOI, PE, PEU, PU, and ATU e-learning systems. This study backs up the amplitude and trajectory of the following direct relationships: PEU, PU, students’ ATU e-learning system, and SSE, which are supported by findings from the main technology acceptance literature (Davis, 1989; Venkatesh et al., 2003) and past e-learning research (Mohammadi, 2015; Abdullah and Ward, 2016; Ching-Ter et al., 2017; Alamri et al., 2020a). This study showed a lot about the Technology Acceptance Model by looking at the students’ ATU e-learning system and SSE, as well as their PU, PE, and PEU. Additional criteria examined in the study were the EDQ, SOI, and SSE, which all have an influence on students’ utilization of the ATU e-learning system. In the current investigation, PEU and PU were shown to have a positive effect on EDQ, SOI, and PE. This discovery is consistent with past research in the field. This implies that before deciding to adopt e-learning programs, students must first assess if they will meet their study demands or be useful in their studies. Students will not believe that e-learning systems are more beneficial until they understand that they are superior to traditional learning without e-learning (Wu and Wang, 2005; Chang and Tung, 2008; Al-Rahmi et al., 2020). Both PU and PEU appear to have a significant influence on ATU’s e-learning system and SSE for students. Given that students’ ATU e-learning system has a favorable impact on SSE of e-learning system usage, it can be inferred that EDQ, SOI, and PE have a positive impact on PEU and PU. In actuality, the e-learning system appears to have a stronger favorable influence on students’ relative faith in EDQ, social power, and physical activity than other systems. This also backs up what (Mohammadi, 2015) discovered in their study, which revealed that EDQ and social effects were the most important elements affecting students’ views about utilizing e-learning systems to improve SSE. According to the findings, information systems (IS) department personnel should focus more on enhancing PEU while not affecting the PU of the system. The significant path coefficient of PU, which has a favorable influence on students’ attitudes and contentment with utilizing an e-learning system, demonstrates this. The researchers observed that the EDQ, SOI, PE, and PEU all had a beneficial effect on PU, which is in line with previous findings (Mohammadi, 2015; Sayaf et al., 2021). Furthermore, the study revealed that EDQ, SOI, and PE all had a beneficial impact on PU and PEU, which is in line with prior research (Mohammadi, 2015; Al-Rahmi et al., 2021a). According to the findings of our study, higher PEU and PU were linked to a higher degree of students’ attitude toward utilizing an e-learning system and satisfaction. As a result, this study demonstrated that EDQ, SOI, and PE all have a direct impact on PEU and PU. These findings are consistent with Davis (1989), Venkatesh et al. (2012), Mohammadi (2015), and Al-Rahmi et al. (2021a). This study included three empirical pieces of proof. These examples were empirical evidence of E-learning system use based on PEU and PU; empirical evidence of students’ ATU e-learning system and SSE through PEU and PU; and empirical evidence of PU and PEU E-Learning systems through EDQ, SOI, and PE that can affect students’ ATU e-learning system and SSE. Therefore, this study contributes to the literature by suggesting a model that assimilates educational quality and social influence factors from constructivism theory with TAM Model, which demonstrated beneficial model to understand the following:

• Educational quality influences perceived ease of use and perceived usefulness in e-learning which increases the positive students’ attitude toward use; thus, it affects students’ satisfaction and academic performance.
• Social influence influences perceived ease of use and perceived usefulness in e-learning which increases the positive students’ attitude toward use; thus, it affects students’ satisfaction and academic performance.
• Perceived enjoyment influences perceived ease of use and perceived usefulness in e-learning which increases the positive students’ attitude toward use; thus, it affects students’ satisfaction and academic performance.
• Perceived enjoyment influences perceived ease of use and perceived usefulness in e-learning which increases the positive students’ attitude toward use; thus, it affects students’ satisfaction and academic performance.
• Perceived ease of use and perceived usefulness in e-learning increases the positive students’ attitude toward use; thus, it affects students’ satisfaction and academic performance.
• Development of theoretical model addressing e-learning usage for education and other related technologies in Saudi higher education.

Additionally, this research contributes first model is integrated two theories constructivism theory, and TAM Model, also helps in application of upcoming e-learning utilize and computer mediated systems which want to implement online learning with the intention of more advantages. Therefore, the major practical implications and contributions of this study were achieved by responding research questions abridged as follows:

• Constructivism theory provided evidence to be a suitable model to understand educational quality and social influence factors for online learning to improve students’ attitude toward use e-learning which in turn increases students’ satisfaction and educational performance in in Saudi higher education.
• Technology acceptance model provided evidence to be a suitable model to understand perceived enjoyment, perceived ease of use and perceived usefulness factors for online learning to improve students’ attitude toward use e-learning which in turn increases students’ satisfaction and educational performance in in Saudi higher education.
• Moreover, human-computer interaction (HCI) has recently tried to analyze users’ behavior for improvement of social technology design, including e-learning. This is consistent with other studies from developed countries such as Kim et al. (2012), Liaw and Huang (2013), Ching-Ter et al. (2017), Krishnan and Hussin (2017), Salloum et al. (2019), Hoq (2020), and Alammary et al. (2021).

### Conclusions, limitations, and work in the future

The current study used an educational context to validate the TAM, EDQ, and SOI, providing fresh information about students’ future opinions on E-Learning system utilization. Furthermore, we assessed the EDQ, SOI, and PE impacting PEU and PU on students’ ATU e-learning system and SSE using an integrated model of the TAM model with EDQ and SOI, and assessed the EDQ, SOI, and PE impacting PEU and PU on students’ ATU e-learning system and SSE using an integrated model of the TAM model with EDQ and SOI. Furthermore, PEU had a favorable impact on students’ ATU e-learning system and SSE, whereas PU had a positive impact on students’ ATU e-learning system and SSE. This study differs from prior studies in the following respects: To begin, this research will incorporate an integrated TAM model of EDQ and social influence into the usage of e-learning. Second, in contrast to other studies in Saudi Arabia, such as Rajab (2018), Alshehri et al. (2019), and Alharbi and Sandhu (2019), this study aims to support a comprehensive review of current articles in the field of e-learning. Third, unlike earlier research that has focused just on the intention to use, this study investigates the impact of many factors on students’ attitudes toward and satisfaction with using an e-learning system. Therefore, the current study is expected to yield a diverse set of findings as well as useful information about students’ views about and satisfaction with using an e-learning system. The results of our study, which took place at two public universities, showed that EDQ, SOI, and PE had the greatest positive impact on PEU, and that PU has an impact on students’ ATU e-learning system and SSE. A moderator analysis was not necessarily due to the limited sample size. Research including many countries, universities, or technologies might give the extra experimental power and data stability needed to look at moderator effects and additional acceptance values. Qualitative research would be necessary to comprehend the similarities and differences between the many views of UTAUT1 and UTAUT2 variables by context. EDQ and its social ramifications Following the establishment and validation of this study’s TAM model, more work is needed to extend the findings to other settings, evaluate the model’s breadth of application, and identify them when applying the model to societally significant technologies. Extending the study to additional technology-related topics including M-loyalty, E-organizational software adoption, and E-readiness, as well as a larger sample size, improves existing IS application utilization findings.

## Data availability statement

The original contributions presented in this study are included in the article/supplementary material, further inquiries can be directed to the corresponding author.

## Ethics statement

Ethical review and approval was not required for the study on human participants in accordance with the local legislation and institutional requirements. Written informed consent from the patients/participants or patients/participants legal guardian/next of kin was not required to participate in this study in accordance with the national legislation and the institutional requirements.

## Author contributions

MMA and WA-R: software. AS, MAA, and WA-R: validation. MAA and WA-R: formal analysis. AS and MAA: project administration and funding acquisition. AS, MAA, MMA, and WA-R: conceptualization, methodology, investigation, resources, data curation, writing—original draft preparation, writing—review and editing, visualization, supervision, read, and agreed to the published version of the manuscript.
